# Does informal care reduce public care expenditure on elderly care? Estimates based on Finland’s Age Study

**DOI:** 10.1186/1472-6963-13-317

**Published:** 2013-08-15

**Authors:** Sari Kehusmaa, Ilona Autti-Rämö, Hans Helenius, Pekka Rissanen

**Affiliations:** 1 Research Department, Social Insurance Institution of Finland, Helsinki, Finland; 2 Department of Biostatistics, University of Turku, Turku, Finland; 3 Tampere School of Public Health, University of Tampere, Tampere, Finland

**Keywords:** Formal care, Informal care, Costs, Public expenditure, Elderly, Long-term care, Health and social services

## Abstract

**Background:**

To formulate sustainable long-term care policies, it is critical first to understand the relationship between informal care and formal care expenditure. The aim of this paper is to examine to what extent informal care reduces public expenditure on elderly care.

**Methods:**

Data from a geriatric rehabilitation program conducted in Finland (Age Study, n = 732) were used to estimate the annual public care expenditure on elderly care. We first constructed hierarchical multilevel regression models to determine the factors associated with elderly care expenditure. Second, we calculated the adjusted mean costs of care in four care patterns: 1) informal care only for elderly living alone; 2) informal care only from a co-resident family member; 3) a combination of formal and informal care; and 4) formal care only. We included functional independence and health-related quality of life (15D score) measures into our models. This method standardizes the care needs of a heterogeneous subject group and enabled us to compare expenditure among various care categories even when differences were observed in the subjects’ physical health.

**Results:**

Elder care that consisted of formal care only had the highest expenditure at 25,300 Euros annually. The combination of formal and informal care had an annual expenditure of 22,300 Euros. If a person received mainly informal care from a co-resident family member, then the annual expenditure was only 4,900 Euros and just 6,000 Euros for a person living alone and receiving informal care.

**Conclusions:**

Our analysis of a frail elderly Finnish population shows that the availability of informal care considerably reduces public care expenditure. Therefore, informal care should be taken into account when formulating policies for long-term care. The process whereby families choose to provide care for their elderly relatives has a significant impact on long-term care expenditure.

## Background

The world’s population is progressively ageing. By 2025, it is estimated that those aged over 65 years will represent 10% of the population, equaling 800 million people globally [1]. This megatrend of ageing will increase the demand for long-term care [2-4]. At the same time, the contribution of family members in elderly care has become increasingly important. Within the European Union (EU), over 80% of all care is provided by family careers [5].

Ageing people naturally prefer to live in their own homes for as long as possible. Informal care enables the elderly to continue to live in the community and to avoid expensive long-term care. If the level of care currently provided by family members decreases in the future, many elderly people will have to leave their communities and enter nursing homes.

Because of its high costs, the use of nursing and residential care has dominated discussions concerning the long-term care of the elderly [6]. To formulate sustainable long-term care policies, it is critical to understand the relationship between the provision of informal care and public expenditure on elderly care. Policy initiatives that encourage family care giving are only cost-effective if informal care does indeed reduce expenditure on elderly care.

Several recent policy initiatives have been proposed to encourage families to provide care to their elderly relatives. In many EU countries, employees have the opportunity to take unpaid leave to care for family members. Many countries offer special services to family careers and some provide families with direct financial assistance to offset the costs associated with elderly care [5]. To evaluate the cost-effectiveness of these initiatives, we require more detailed information concerning the relationship between informal care and formal care expenditure on elderly care.

There is a large volume of published studies that describe the role of informal care provided to the elderly. The generalizability of such research results depends on the definition of informal care. The definition of informal care can include help in tasks related to the activities of daily living (ADL), such as dressing, bathing, eating and using the toilet, or in instrumental activities of daily living (IADL), such as preparing hot meals, shopping for groceries, taking medication or managing money. Sasso et al. (2002) found that informal care reduced the probability of nursing home entry when it included help with ADL tasks, but no significant reduction in the likelihood was found when the help was measured more broadly to include, for example, help in preparing meals or shopping.

It is unclear whether caring for close relatives, friends or neighbors actually serves as a substitute of formal care and assistance. If it is indeed a substitute, it means that such informal care will decrease the use of formal services, and as a result, reduce public long-term care expenditure. However, informal care can also complement formal services, and as such, formal care is required regardless of the informal care received.

Previous studies have analyzed the effect of informal care on the use of formal care. The results are mixed. Examining the hypothesis of mixed responsibility, Motel-Klingebiel et al. (2005) suggested that the total quantity of assistance received by older people is greater in welfare states with a strong formal services infrastructure. In other words, they found no evidence of a substantial ‘crowding out’ of family help by the extensive provision of formal services [7].

In contrast, other studies found a negative correlation between the provision of informal care and the use of formal services. This view is supported by Stabile et al. (2006). They found that the increased availability of publicly financed home care is associated with an increase in its utilization and a decline in informal care giving. Viitanen et al. (2007) found a similar substitute effect in Europe. According to their results, an increase of 1,000 Euros in the public expenditure on formal residential care and home help services for the elderly decreased the probability of informal care outside of the caregiver’s household by 6 percentage points [8].

The relationship between informal care and different types of formal care varies. Van Houtven and Norton (2004) found a net substitution for all types of care [9]. In addition, Bolin et al. (2008) found that informal care is a substitute for formal home care, but is a complement to doctor and hospital visits [10]. There is also a relationship between the level of disability and informal care [11]. Those in the poorest health require formal services regardless of the available informal care.

Co-residence with the caregiver has an influence on the total quantity of assistance received [7]. If key supporters share the same household with the subject, they are more likely to provide support every day (96%) compared with those who were not living in the same household (36%) (p < 0.001). Key supporters are generally found to be spouses (38%), daughters (30%) and sons (9%) [12].

Less research has been conducted on the economic aspects of the impact of informal care on formal care expenditure. Based on previous research, the functional ability of the elderly person should be taken into account when estimating expenditure. Earlier studies have shown that physical and cognitive health problems increase both the probability of receiving informal care and the probability of institutionalization [6].

The objective of our research is to examine the effects of informal care on public care expenditure for frail elderly persons. Using data obtained from the Age Study, conducted nationwide in Finland, we modeled the costs of care in four care patterns over a 1-year period for a sample of elderly Finnish people. For the modeling, we first used a set of multilevel regression analyses to identify which variables are associated with the use and costs of health and social care. Second, we used the effects thus found to adjust the mean formal care costs in the four care patterns. The alternative care patterns are: 1) informal care only for elderly living alone; 2) informal care only from a co-resident family member; 3) a combination of formal and informal care; and 4) formal care only. Our hypothesis is that informal care reduces public expenditure on elderly care.

## Methods

### Sample

The data were sourced from a geriatric rehabilitation program for frail elderly persons conducted from 2002 to 2007 in Finland (Age Study) [13]. The inclusion criteria were persons aged 65+ years, with progressively decreasing functional ability, and at risk of institutionalization within two years. The definition of frailty is based on the entitlement criteria for the Pensioners’ Care Allowance benefit granted by the Social Insurance Institution of Finland (SII). This definition covers biological, physiological, social and environmental changes. The subjects were enrolled through a two-phase selection process. In the first phase, potential participants were recruited by local social and health care officials in 41 municipalities. In the second phase, representatives of the relevant municipality, rehabilitation center and local SII office jointly assessed the selected candidates’ eligibility and suitability for rehabilitation. Our analysis is based on a sample of 732 frail elderly persons living in 41 municipalities (Table 1).

**Table 1:** Distribution of care patterns according to the characteristics of participants in the Age Study

	**All**		**Informal care only for elderly living alone**		**Informal care only from a co-resident family member**		**A combination of formal and informal care**		**Formal care only**		**Missing care information**		**P-value of** **Chi-squared test**
	**(n = 732)**		**(n = 184)**		**(n = 151)**		**(n = 337)**		**(n = 45)**		**(n = 15)**		
**Variable**	**n/mean**	**%**	**n/mean**	**%**	**n/mean**	**%**	**n/mean**	**%**	**n/mean**	**%**	**n/mean**	**%**	
**Age group**													
65–74	215	30	51	28	71	47	75	22	14	31	4	27	
75–84	356	49	95	52	63	42	169	50	20	45	9	60	
85+	161	21	38	20	17	11	93	28	11	24	2	13	<0.0001
**Gender**													
Male	101	14	16	9	34	23	40	12	11	25	0	0	
Female	631	86	168	91	117	77	297	88	34	75	15	100	0.0003
**Financial situation**													
Good	141	19	28	15	31	20	71	21	7	16	4	27	
Average	502	69	138	75	98	65	228	67	31	68	7	46	
Poor	89	12	18	10	22	15	38	11	7	16	4	27	NS
**Self-assessed health**													
Good	29	4	6	3	10	7	12	4	1	2	0	0	
Average	477	65	116	63	100	66	220	65	29	65	12	80	
Poor	226	31	62	34	41	27	105	31	15	33	3	20	NS
**IADL**													
Good	170	23	79	43	37	25	43	13	7	16	4	27	
Medium	402	55	96	52	72	48	201	60	23	51	10	67	
Poor	160	22	9	5	42	27	93	27	15	33	1	6	<0.0001
**FIM™ score§**													
Limited physical function score <120	449	61	79	43	71	47	252	75	37	82	10	67	<0.0001
GDS score*													
Depressive mood score >7	74	10	14	8	11	7	41	12	6	13	2	13	NS
**MMSE score†**													
Declined cognitive capacity score <24	210	29	40	22	30	20	119	35	17	38	4	27	0.0007
**Mean HRQoL 15D‡**	0.73		0.76		0.74		0.72		0.72		0.75		0.0013

§ FIM: Functional Independence Measure, maximum score 126, three subscales (Self Care, 8 items; Mobility, 5 items; Cognition 5 items) were formed from 18 items (range: 1 = total assistance–7 = complete independence).

* GDS: Geriatric Depression Scale, maximum score 15, values 0–6 indicate non-depressive state.

† MMSE: Mini Mental State Examination, maximum score 30, values under 24 indicate existence of dementia.

‡ 15D: Health-related quality of life (HRQoL), range 0–1, 1 indicates the best imaginable health.

Information regarding the participants’ use of services was gathered through self-reported questionnaires and register data. Functional assessments were conducted by three physiotherapists. Register data on the utilization of health and social care services were obtained from the national databases of the Care Registers for Social Welfare and Health Care [National Institute for Health and Welfare (THL), formerly Stakes] [14] and SII [15].

The Age Study was approved by the Ethical Committees of the SII and Turku University Hospital. All of the study participants gave their written consent to the study.

### The framework for health care and social services in Finland

In Finland, the national targets for services for people aged over 75 years old are as follows: 92% will live at home independently or use appropriate health and welfare services; 14% will receive regular home care; 5%–6% will receive informal care support; and 8%–9% will live in sheltered housing with 24-hour assistance or in long-term care in health center hospitals [16].

Family members are an important source of care and assistance for older people. The municipality can support the informal caregiver by paying a specific fee for the care they provide and/or by arranging a range of social welfare and health services that support the care giving.

### Study variables

#### Care patterns

Our analysis focused on the public care expenditure in four care patterns over a 1-year period. The relevant care categories were formed on the basis of earlier studies [7,9-12,17], taking into account co-residence with an informal caregiver and possible mixed responsibility between family care and formal care. The four care patterns are: 1) informal care only for elderly living alone; 2) informal care only by a co-resident family member; 3) a combination of formal and informal care; and 4) formal care only.

In our study, informal care is defined to include those tasks that have a counterpart in formal care and will therefore have an effect on public expenditure on elderly care. Because of this definition, we only took into account the most burdening portion of informal care. Our study thus underestimates the total amount of informal care because it does not include all of the tasks that family members do for the elderly (e.g., shopping, managing money, and companionship). We only included tasks that are substitutes for institutional care, formal home help or home nursing.

To categorize the subjects into the different care patterns, we collected data from various sources. First, municipal social and health care officials were asked to collect information regarding informal and formal care from individual care and service plans. Second, we used self-reported questionnaire data to double-check the data provided by the municipal officials. The baseline assessments were used to categorize the subjects. There were 15 subjects in the sample that we were not able to classify into any of the given care patterns; they were excluded from the analyses.

#### Background variables

The socio-demographic background variables used in this study are as follows: age (categorized in three groups: 65–74, 75–84 and over 85 years), gender, self-assessed financial situation (three categories: Good, Average, Poor) and place of residence.

#### Health and functional ability

To assess functional independence, we used the Functional Independence Measure (FIM) score. The scores range from 18 (lowest level of independence) to 126 (highest level of independence) [18]. Depression was measured by the Geriatric Depression Scale (GDS), with a maximum value 15; values 0–6 indicate non-depressiveness [19]. Cognitive capacity was measured by the Mini Mental State Examination (MMSE), with a maximum value of 30; values under 24 indicate the existence of dementia [20]. The ability of the subject to perform instrumental activities of daily living was measured by the IADL index. We categorized the IADL index into three classes: Good (a score less than 10), Medium (between 10 and 15) and Poor (over 15).

Self-assessed health status was measured by asking the question “How do you perceive your health at present?” The three classes of this variable were Poor (included responses “very poor” or “poor”), Average (“average”) or Good (“good” or “very good”). The validity of single item measures has been discussed in the relevant literature. There is evidence that a measure containing a single, global question is likely to be appropriate, rather than a multi-item measurement scale. Single item measures have been judged to be suitable for use in population surveys [21]. Health-related quality of life (HRQoL) was evaluated by the 15D score, with a range of 0–1, where 1 indicates the best imaginable health [22].

#### Rehabilitation

In the Age Study, the subjects were randomly assigned either to an in-patient rehabilitation program or to standard care. Thus, to standardize the effect of rehabilitation, we included rehabilitation as an explanatory variable in all our models.

#### Formal care expenditure

The utilization of health care services and medicines during the 1-year period was assessed on the basis of data derived primarily from national health care registers. Data on inpatient care and day stay surgery were collected from the national databases of the Care Registers for Social Welfare and Health Care (THL). Data on outpatient care within the private sector and the use of medicines were obtained from SII registers. A self-reported questionnaire was used to collect information from the subjects on their use of public sector outpatient care because there is no available register data.

Utilization of social services covers institutional care and professional home care. For those living in residential homes and sheltered housing, services such as home help, washing and cleaning were included. For those living at home, professional home care, home nursing, and support services were included. Data on the utilization of social care services were obtained from the questionnaires. We asked the municipal social and health care officials to collect service use data from their clients’ individual care and service plans. The data derived from questionnaires were cross-sectional both at baseline and in the 12-month follow-up. For those cases where changes occurred in the use of services during the follow-up, the annual data comprised 6 months of services received at the baseline and 6 months of services received at the follow-up.

Formal care expenditure was determined by multiplying the frequency of use of services by their average unit costs. For the monetary valuation of the health and social care services, we used Finnish standard costs information [23]. The price year was 2010 and the currency was the euro.

### Data analysis

The data analysis proceeded in two stages. First, we estimated the effect of the explanatory variables on the formal care expenditure using the following four models (Table 2):

MODEL 1 = Rehabilitation + Care Pattern + Background variables; random effect: municipality

MODEL 2 = Rehabilitation + Health and functional ability + HRQoL; random effect: municipality

MODEL 3 = Rehabilitation + Care Pattern + Functional ability + HRQoL; random effect: municipality

MODEL 4 = All variables; random effect: municipality

**Table 2:** **Results of regression analysis showing the regression coefficients (β) with 95**% **confidence intervals (CI) and p-values for logarithm-transformed public expenditure on care**

	**MODEL 1=Rehabilitation + Care + Background variables**					**MODEL 2 = Rehabilitation + Health and Functional ability + Health-related Quality of Life**					**MODEL 3 = Rehabilitation + Care + Functional ability + Health-related quality of life**					**MODEL 4 = All variables**				
	**MODEL 1**					**MODEL 2**					**MODEL 3**					**MODEL 4**				
**Variable**	**β**	**(CI 95%)**		**p-value**		**β**	**(CI 95%)**		**p-value**		**β**	**(CI 95%)**		**p-value**		**β**	**(CI 95%)**		**p-value**	
**Intercept**	9.14	8.75	9.54	<0.0001	*	14.37	12.58	16.15	<0.0001	*	13.40	12.09	14.71	<0.0001	*	12.60	10.91	14.29	<0.0001	*
**Rehabilitation**																				
Yes	0.05	−0.11	0.21	0.5587		0.15	−0.02	0.33	0.0895		0.09	−0.07	0.25	0.2728		0.09	−0.07	0.25	0.2858	
No	0					0					0					0				
**Care received**																				
Informal care only for elderly living alone	−1.34	−1.70	−0.97	<0.0001	*						−1.21	−1.58	−0.84	<0.0001	*	−1.15	−1.53	−0.77	<0.0001	*
Informal care only from a co-resident family member	−1.54	−1.92	−1.16	<0.0001	*						−1.44	−1.81	−1.06	<0.0001	*	−1.42	−1.80	−1.04	<0.0001	*
A combination of formal and informal care	−0.07	−0.42	0.28	0.7081							−0.13	−0.48	0.22	0.4529		−0.10	−0.46	0.25	0.5628	
Formal care only	0										0					0				
**Age group**																				
65–74	−0.04	0.74	−0.29	0.2016												−0.01	−0.26	0.23	0.9157	
75–84	0.12	0.28	−0.10	0.329												0.07	−0.14	0.28	0.5294	
85+	0															0				
**Gender**																				
Male	0.18	−0.07	0.43	0.1554												0.14	−0.11	0.39	0.2571	
Female	0															0				
**Financial situation**																				
Good	−0.12	−0.33	0.10	0.2937												−0.10	−0.32	0.11	0.3588	
Average	0															0				
Poor	0.09	−0.17	0.35	0.501												−0.09	−0.35	0.17	0.5014	
**Self-assessed health**																				
Good						−0.06	−0.58	0.45	0.8095							−0.16	−0.63	0.31	0.4992	
Average						−0.05	−0.27	0.18	0.6873							−0.17	−0.37	0.03	0.0956	
Poor						0										0				
**IADL**																				
Good						−0.51	−0.83	−0.19	0.0019	*						−0.34	−0.65	−0.04	0.0269	*
Medium						−0.16	−0.41	0.09	0.1981							−0.19	−0.42	0.04	0.1029	
Poor						0										0				
**FIM™ score**						−0.04	−0.05	−0.03	<0.0001	*	−0.03	−0.04	−0.02	<0.0001	*	−0.02	−0.04	−0.01	0.0001	*
**GDS score**						0.03	−0.01	0.07	0.1796							0.00	−0.03	0.04	0.8403	
**MMSE score**						−0.03	−0.06	0.00	0.068							0.00	−0.03	0.03	0.9132	
**HRQoL 15D score**						−0.10	−1.63	0.64	0.3918		−1.16	−2.01	−0.30	0.0079	*	−0.42	−1.45	0.62	0.4286	
* (p < 0.05)																				

We examined bivariate correlations between independent variables to check for correlations. All correlations were low (<0.5). Multilevel modeling with fixed (patient level) and random (municipality level) effects was used to estimate the effects of explanatory variables on the public care expenditure [24]. SAS PROC MIXED was applied to fit the multilevel model [25].

All four models were adjusted for rehabilitation to standardize the effect of the rehabilitation in the original randomized trial setting. Care expenditure was analyzed to rule out skewness, and logarithm transformation was used. The effect sizes, as the result of linear analyses, were expressed as estimates with 95% confidence intervals (CI) and the corresponding p-values (Table 2).

Second, we calculated the average care expenditure in the four care patterns. We adjusted the mean expenditure by the effects that were found to be associated with expenditure in the regression analysis. The expenditure was calculated according to the formula:

$$\begin{array}{l} {\text{Expenditure}}_{\left(\text{Total}\right)}=\sum_{\left(c,f,h\right)}{\text{Expenditure}}_{\left(\text{Social care}\right)}\\ \;+\sum_{\left(c,f,h\right)}{\text{Expenditure}}_{\left(\text{Health care}\right),} \end{array}$$

where

c = care pattern,

f = functional independence, and

h = HRQoL.

Table 3 shows the estimation results. For the log-transformed data, we used Smearing estimates to retransform them back to euro values [26]. In addition, we used a basic service price index to discount the expenditure. The data were analyzed with LS-means from PROC MIXED SAS 9.1.

**Table 3:** Estimates of the public care expenditure in four care patterns

	**Estimate of public care expenditure, adjusted for FIM and HRQoL 15D**				
**Care received**	**Logarithm-transformed expenditure**	**(CI 95%)**		**Smearing estimates of expenditure**	**Expenditure discounted to price year 2010**
Informal care only for elderly living alone	8.00	7.83	8.17	4 600	6 000
Informal care only from a co-resident family member	7.76	7.58	7.95	3 800	4 900
A combination of formal and informal care	9.07	8.94	9.20	17 200	22 300
Formal care only	9.21	8.88	9.55	19 500	25 300

Within-group mean logarithm transformed expenditure with 95% confidence intervals (CI) adjusted for functional independence (FIM) and health-related quality of life (HRQoL 15D).

Corresponding Smearing estimates of expenditure, and expenditure discounted to the price year 2010 with a basic service price index.

## Results

Table 1 shows the characteristics of the sample (n = 732). The subjects’ mean age was 78 years (range 65–96 years). The majority were female (86%) and had limited physical functioning capacity (61%). Depressive mood was detected in 10% of the sample, 29% had declined cognitive capacity and 22% poor IADL skills.

Differences were found in level of disability among the four care patterns. Limited physical functioning capacity was detected more often among subjects receiving either formal care only or a combination of formal and informal care. IADL skills were more likely to be higher if the subject was living alone. Furthermore, the proportion of subjects receiving formal care was higher in the male population. Informal care by a co-residing person was more common for those aged 65–74 years.

### Total expenditure

The results of the multivariate regression analyses are presented in Table 2. The level of informal care (“Care received”) was associated with public care expenditure. In the first model, we used background variables as explanatory variables, and found that the care pattern was the only variable that was significantly associated with care expenditure (Model 1). In the second model, we used several aspects of functional ability and HRQoL to predict expenditure. IADL skills and FIM scores were associated with expenditure on care (Model 2). An earlier study of ours has shown that FIM is associated with social care service use, and HRQoL 15D appears to be a powerful indicator for the utilization of health care services [27]. Based on this prior knowledge, in Model 3, public care expenditure was controlled for independent disability level (FIM) and health-related quality of life (HRQoL 15D) [27]. These results show that FIM, HRQoL15D and Care Pattern are associated with expenditure (Model 3). Finally, Model 4 is a fully adjusted model (Model 4).

Table 3 presents the adjusted mean expenditure of care for the four different care patterns. Model 3 was used to adjust the care patterns. Based on the regression analysis, we adjusted for the subject’s functional status and health state by using FIM and 15D as explanatory variables and municipality as the random variable. When the patient structure is standardized to be the same in all four care patterns, the expenditure was found to be the highest (25,300 Euros/year) when formal care was the only source of care and assistance. For the combination of formal and informal care, the expenditure amounted to 22,300 Euros/year. The least expensive way to organize care was via informal care provided by a co-resident family member (4,900 Euros/year). Living alone increased care costs, but they were still low (6,000 Euros/year). The public care expenditure for those who received only informal care included both health care costs and service costs that support family care giving.

## Discussion

Our research shows that the availability of informal care significantly reduces public expenditure on care of the frail elderly. When formal care was the only source of care, the annual mean expenditure of care was the highest (25,300 Euros). Informal care from a co-resident family member reduced the annual mean expenditure by a total of 20,400 Euros (to 4,900 Euros), and correspondingly, for a person living alone by 19,300 Euros (to 6,000 Euros annually).

The cost implications of our findings are significant because informal care is commonly used to care for the elderly. In Finland, approximately 140,000 elderly people aged over 70 years receive informal care [28]. We found that the estimated mean savings in public care expenditure from informal care is 20,000 Euros/person per year. In total, informal care reduces the annual expenditure of elderly care by approximately 2.8 billion Euros. Without informal care, public care expenditure would be two times higher than at present.

Our results are of significance for policy initiatives designed to promote family care. In Europe, informal care is essential in terms of the sustainability of long-term care systems. Public funding does not cover the contributions made by family members. However, most countries have policies to support informal caregivers. They either provide cash benefits to carers or offer services aimed to support informal care. For evaluating the cost-effectiveness of these initiatives, our study provides empirical knowledge of the extent to which informal care actually reduces public care expenditure.

In our study, informal care by a co-residing caregiver was more likely in the 65–74 years age group. In that age group, the co-resident caregiver is often the spouse. Previous studies have shown that the presence of a spousal caregiver increases care hours, but does not affect nursing home entry [29]. We found that a co-resident caregiver effectively reduced care expenditure (co-resident caregiver versus formal help users, p < 0.0001). There is, however, a relationship between the level of the patient’s disability and the burden of the informal caregiver [11]. Those in the poorest health often need various formal services. The risks of adverse effects on the caregivers’ own health and well-being also increase with the level and intensity of the formal care provided [11].

It is obvious that the physical health of the elderly has to be taken into account when researching the impacts of informal care. In our study, those who received formal care only were more likely to have limited physical functioning, declined cognitive capacity and reduced HRQoL. We included a FIM and HRQoL 15D into our models. As evidenced in one of our previous studies [30], HRQoL is a strong predictor of health costs and FIM is related to social care costs. These measures are related to the caregiver’s burden and workload, and this method standardizes care needs within a heterogeneous subject group. This approach enabled us to compare expenditure among the various care categories even when differences in physical health were observed.

Our result is a conservative estimate of the extent to which informal care reduces public expenditure on elderly care. To control for selection bias we calculated LS means, which are predicted population margins, estimating the marginal means over a balanced population. Our results are consistent with Bonsang’s (2009) earlier findings that informal care is a substitute for paid domestic help and nursing care, but the substitution effect tends to disappear as the level of disability of the elderly person increases.

Because more than half of caregivers are retired, the impact of informal care giving on the labor force was minimal in our study. Previous studies have shown, however, that informal care may affect labor force participation [31,32]. Regarding working-age caregivers, the flexibility of the labor market allows people to choose between work and caring. The right to choose is seen as a main indicator of welfare [33].

Further research is required to investigate the process by which families choose to provide care for their elderly relatives. An increase in the number of single-living households and unmarried people will reduce the availability of caring spouses. A decline in the number of children may also reduce the availability of future care to elderly parents.

Our objective was to study the financial impact of informal care on public care expenditure, which we consider is important for policymaking. The result is not an estimate of the total monetary value of informal care because we limited our analysis to care that serves as a substitute for formal care. In other studies, the monetary value of informal care is usually based on valuing the caregivers’ time input (hours of caring provided). These studies vary regarding what is included in informal care and how the hours of caring are priced.

The strength of our study is the use of a nationwide population-based sample. The majority of expenditure was calculated using Finnish register data, which are regarded as very reliable. In addition, we did not limit the analysis to any single type of formal care, but included all social services and health care usage in the expenditure.

## Conclusions

In conclusion, our analysis of a sample of a frail elderly Finnish population shows that the availability of informal care has a major impact on reducing public expenditure in elderly care, and therefore informal care should be taken into account when formulating policies for long-term care.

## Competing interests

The authors have no financial or non-financial competing interests.

## Authors' contributions

SK participated in the design and coordination of the study, performed the statistical analysis and drafted the manuscript. IA-R participated in the design and coordination of the study and drafted the manuscript. HH tutored and participated in the statistical analysis and helped draft the manuscript. PR participated in the design of the study and drafted the manuscript. All authors have read and approved the final manuscript.

## Pre-publication history

The pre-publication history for this paper can be accessed here:

http://www.biomedcentral.com/1472-6963/13/317/prepub
